# MiRNA‐96‐5p impacts the progression of breast cancer through targeting FOXO3

**DOI:** 10.1111/1759-7714.13348

**Published:** 2020-02-26

**Authors:** Ziyi Yin, Wenyan Wang, Gengbao Qu, Lin Wang, Xiang Wang, Qin Pan

**Affiliations:** ^1^ Department of Breast Surgery Beijing Tiantan Hospital, Capital Medical University Beijing China; ^2^ Department of Medical Oncology Xuzhou Central Hospital Xuzhou China; ^3^ Department of Oncology Liyang People's Hospital Liyang China

**Keywords:** Breast cancer, FOXO3, miRNA‐96‐5p, proliferation

## Abstract

**Background:**

Breast cancer is the most common malignant tumor in women worldwide, with a high mortality rate. MicroRNAs are small non‐coding RNAs that negatively regulate the expression of target genes by interacting with the target gene 3'‐UTR, and participate in cell differentiation, proliferation, apoptosis and metabolism. The function of miRNA‐96‐5p in the progression of breast cancer has not been reported.

**Methods:**

We used the StarBase database to investigate the expression of miRNA‐96‐5p in breast cancer and adjacent normal tissues. FOXO3 3'‐UTR construct and luciferase reporter assays was performed for the target gene. Expression levels of miRNAs including its target were analyzed by qRT‐PCR and western blot. Cell proliferation was detected by CCK8 and colony formation, EdU assay.

**Results:**

Luciferase reporter assays showed miRNA‐96‐5p directly targeted FOXO3. Abrogation of miRNA‐96‐5p by transfection with its inhibitors in breast cancer cells significantly suppressed miRNA‐96‐5p expression and breast cancer cells proliferation. Western blot revealed that overexpression of miRNA‐96‐5p substantially reduced FOXO3 protein expression. We used the GEPIA, UALCAN and KM‐plotter databases to investigate the expression of FOXO3 in human breast cancer and adjacent normal tissues, and its correlation with survival. In addition, we found that FOXO3 spoiled miR‐96‐5p induced breast cancer cell proliferation block effecting.

**Conclusions:**

miRNA‐96‐5p may exert a tumor promotion role through negatively regulating tumor suppressor gene FOXO3 and promoting cell proliferation.

## Introduction

Breast cancer is the most common malignant tumor in women.^1^ There are approximately 1.3 million new cases worldwide each year, and 500 000 die from breast cancer.^2^ In developed countries such as Europe and the United States, the incidence of breast cancer ranks first among female malignancies.^3^, ^4^ The incidence of breast cancer is increasing year by year, and the age of onset is now being found in women of a much younger age.^5^ It has become the first killer threatening the health of women. The occurrence of breast cancer is a complex biological process caused by multiple genes and multiple factors. With the development of molecular biology, through the analysis of breast cancer gene expression profiles, different gene expressions are related to various biological behaviors of breast cancer. Advances in diagnostic methods have refined the classification of breast cancer and the combined use of multiple treatment methods, making breast cancer treatment more individual.^6^ Therefore, the study of tumor gene profiling is helpful for tumor diagnosis and treatment, and judgment of prognosis.

MicroRNA is a non‐coding single strand RNA consisting of 22 nucleotides.^7^ Bioinformatics analysis confirms there is high evolutionary conservation of miRNA sequence among species. MicroRNAs interact with 3′ non‐coding region (3'‐UTR) of mRNA to promote mRNA degradation, thereby inhibiting mRNA translation.^8^ MicroRNA plays a very important role in cell growth and development, differentiation, proliferation, apoptosis, cell cycle and other cell biological behaviors in the regulation of gene expression. In recent years, an increasing number of studies have found that miRNAs are abnormally expressed in the serum and tumor tissues of cancer patients.^9^ Further studies have shown that miRNAs play an important role in the occurrence and development of tumors. The family miRNA‐183 consists of three miRs: 183, 96 and 182. These miRNAs are concurrently expressed during development. As a member of the miRNA‐183 family, miRNA‐96 is highly conserved in evolution and is located on chromosome 7.^10^ FOXO3 is a member of the forkhead transcription factors and is characterized by a unique forkhead domain.^11^ The abnormal expression of FOXO3 is obviously correlated with the occurrence and development of tumors.^12^ The 3'‐UTR of FOXO3 has two sequence‐conserved binding sites for miRNA‐96, which inhibits the expression of FOXO3 after binding.^13^ Iwai *et al*. confirmed that miRNA‐96 is highly expressed in human hepatocellular carcinoma, and inhibiting miRNA‐96 can increase the expression levels of FOXO1 and FOXO3, thereby reducing the proliferation and colony formation of hepatoma cell lines.^14^ Zhu *et al*. used 70 pairs of NSCLC tissues and matched paracancerous tissues and 44 normal human serum samples to verify that the miRNA‐183 family (including miRNA‐96) is highly expressed in cancer tissues and serum.^15^ MiRNA‐96 has been reported in tumor tissues and serum, but there are few studies focused on its downstream targets. Therefore, this study investigated the effects of miRNA‐96 on human breast cancer cell proliferation and apoptosis.

## Methods

### Cells and reagents

The human breast cancer cell lines MCF‐7 and T47D were purchased from ATCC (Manassas, VA, USA). The breast cancer cells were cultured in DMEM high glucose medium containing 10% fetal bovine serum (Gibco, New York, USA). All cells were incubated at 37°C in a 5% CO_2_ incubator.

### Kaplan‐Meier plotter analysis

The correlation between FOXO3 gene expression and breast cancer prognosis was analyzed using the Kaplan‐Meier database, which integrates gene expression data and clinical data. Our analysis was focused on overall survival (OS) and relapse‐free survival (RFS) patient information.

### Western blotting

Cells were harvested and lysed using RIPA buffer, then mixed with 1x loading buffer. Samples were separated by 10% SDS‐PAGE gel electrophoresis and transferred to PVDF membranes (Millipore, Billerica, MA, USA). The membrane was blocked with 5% skim milk at room temperature for one hour, incubated with primary antibody at 4°C overnight, and then incubated with secondary antibody for one hour at room temperature. Protein bands were visualized by SuperSignal chemiluminescent substrate (Millipore, Billerica, MA, USA). GAPDH and ACTB are used as load internal reference. The antibodies used for immunoblotting were as follows: GAPDH (ab8245) (Abcam), FOXO3 (ab70315) (Abcam), ACTB (3700) (Cell Signaling Technology).

### Quantitative RT‐PCR

Total RNA was extracted from cells using TRIzol (TaKaRa, Dalian, China) according to the manufacturer's instructions. For reverse transcription (RT) of mRNA or microRNA, we used PrimeScript RT master mix (TaKaRa in Japan) or miScript reverse transcription kit (Qiagen, Germany). The specific primers were used for amplification. SYBR Green quantitative PCR reaction was carried out in a 20 μL reaction volume containing 2× PCR Master Mix (Applied Biosystems). The relative quantification of gene expression for each sample was conducted using the ΔΔCт method. Each experiment was performed in triplicate.

### CCK8 assay

We adjusted cell density at 20 000 cells/mL by culture medium supplemented with 10% FBS, and dispensed 100 μL of cell suspension (20 000 cells/mL) into a 96‐well plate. Cell proliferation was detected with a Cell Counting Kit‐8 (MCE, America). Then, 10 μg of CCK‐8 solution was added into each well after culture for 24, 48 and 60 hours, respectively. Optical density values were measured with a microplate reader at an absorbance of 450 nm.

### Colony formation assay

The cells were collected and seeded into a 6‐well plate, with 500 cells per well, and three replicate wells were also set. The cells were incubated for several hours in a 37°C CO_2_ incubator before attaching them to the plate. The cells were then incubated in a CO_2_ incubator at 37°C for 15 days until the cells in the control plates formed colonies of significantly better size (50 cells per colony was the minimum fraction).

### EdU proliferation staining

The proliferative capacity of tumor cells was determined using a Click‐iT EdU cell proliferation assay (RIBOBIO, C10310‐1), and 500 μL of Click‐iT reaction mixture was added to each well and the plate shaken briefly to ensure thorough mixing. The cells were stored in the dark for 30 minutes at room temperature. The reaction mixture was discarded and each well was washed once with 1 mL of PBS containing 3% BSA. The supernatant was discarded and the nuclei stained with DAPI for five minutes at room temperature.

### Luciferase‐reporter activity assay

We cloned wild‐type and mutant MiRNA‐96‐5p binding sequences from FOXO3 3′UTR into the pGL3 Basic vector (Promega, Madison, Wisconsin). MiRNA‐96‐5p and the 3′UTR (wild or mutant) of the FOXO3 recombinant plasmid were cotransfected into 293T cells. Transfection was performed using Lipofectamin 3000 reagent (Invitrogen), and luciferase activity was detected using a dual luciferase reporter detection system (Promega, Madison, Wisconsin).

### Statistical analysis

All data are shown as mean ± standard deviation (SD), and data are calculated based on at least three independent experiments. Student's *t*‐test was used to compare quantitative variables. If the *P*‐value does not exceed 0.05, the data was considered as significant.

## Results

### MiRNA‐96‐5p suppresses the proliferation ability of breast cancer cells

**Figure 1:**
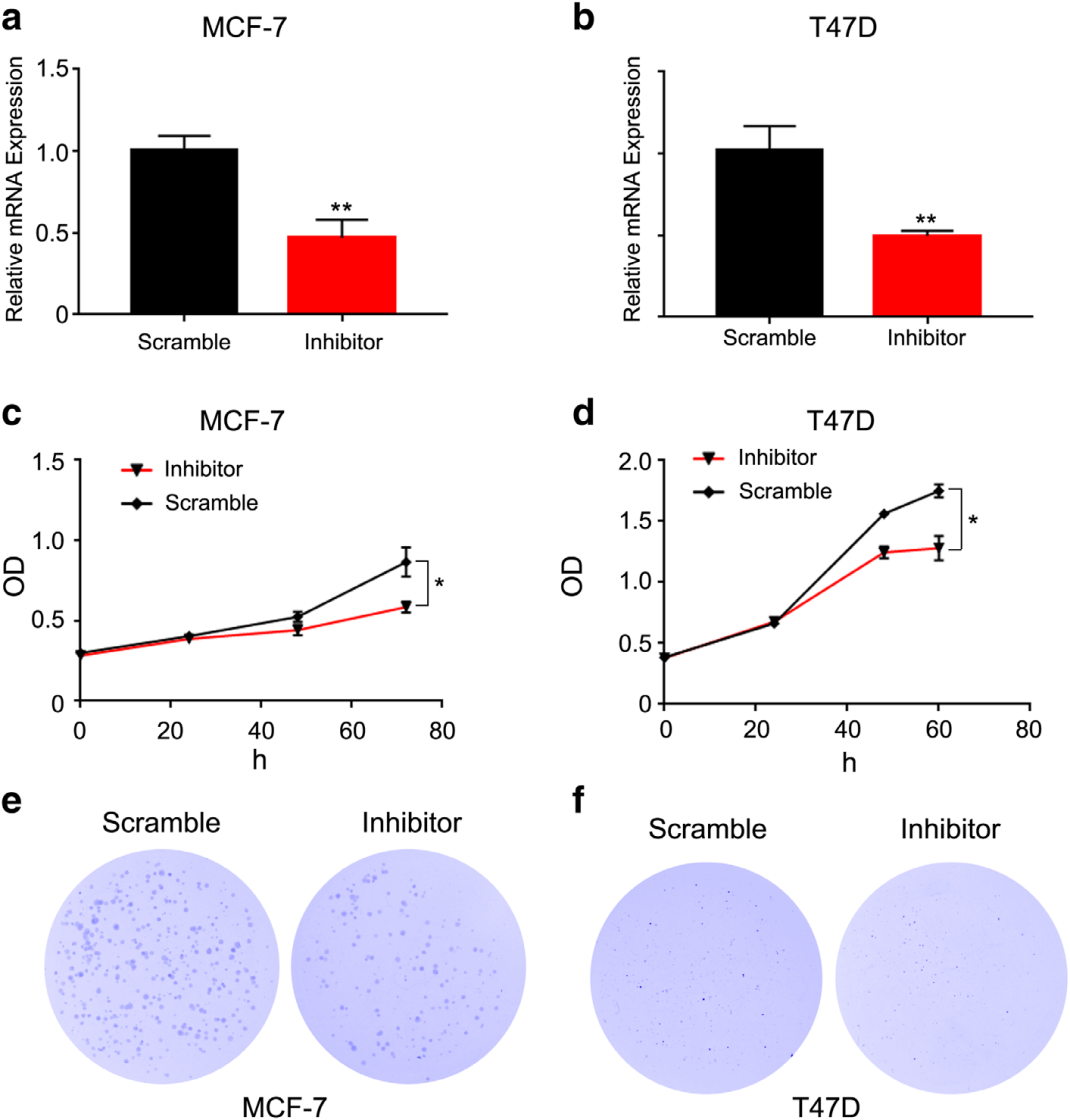
MiRNA‐96‐5p suppressed the proliferation ability of breast cancer cells. (**a**) qRT‐PCR assays showed that MiRNA‐96‐5p expression obviously reduced by transfection of MiRNA‐96‐5p inhibitor into MCF‐7 cells. (**b**) qRT‐PCR assays showed that MiRNA‐96‐5p expression obviously reduced by transfection of MiRNA‐96‐5p inhibitor into T47D cells. (**c**,**d**) Proliferation curves of scramble and inhibitor transfection into MCF‐7 and T47D cells. (**e**,**f**) Colony formation assays of scramble and inhibitor transfection into MCF‐7 and T47D cells.

**Figure 2:**
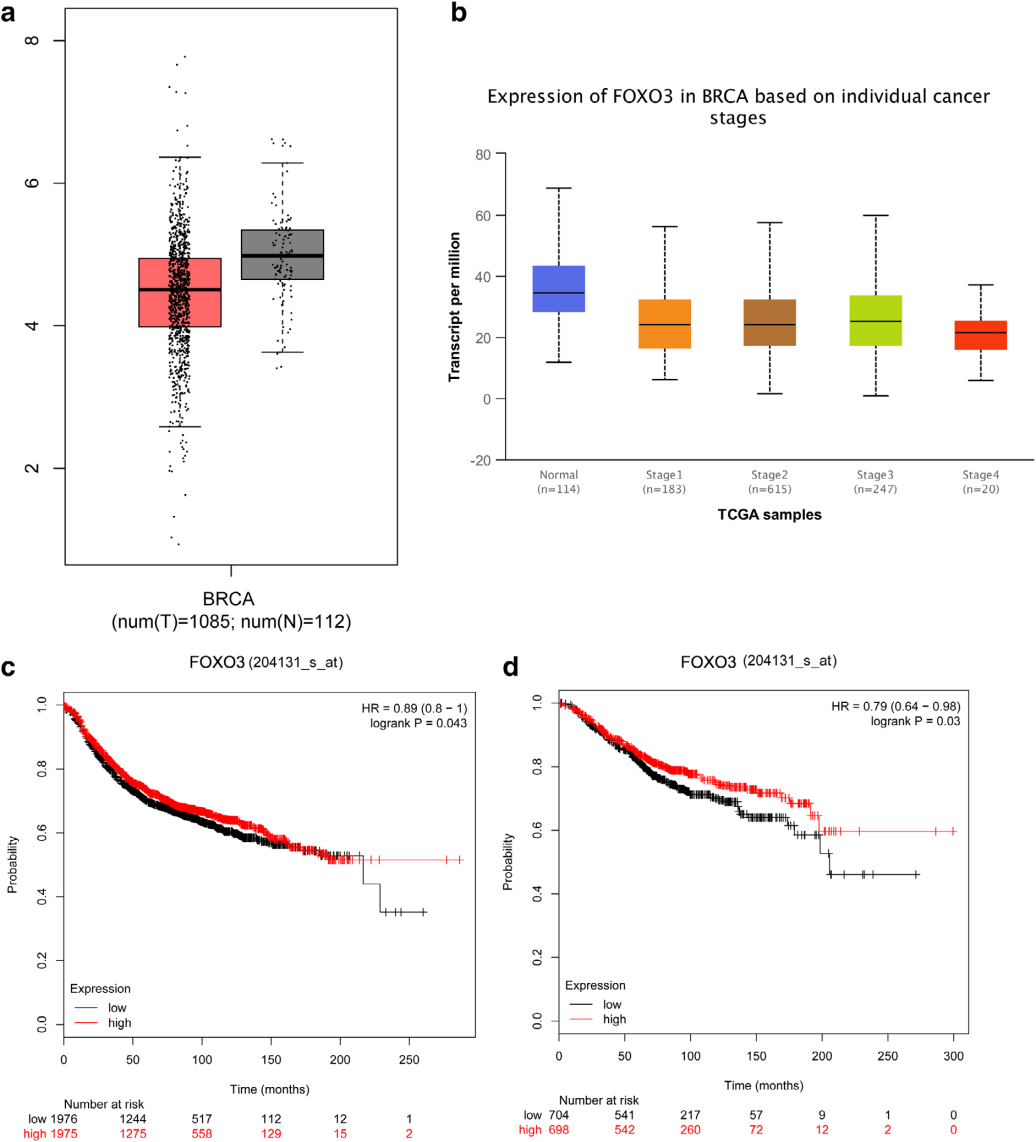
FOXO3 is downregulated in breast cancer and correlated with the prognosis. (**a**) Low FOXO3 expression in breast cancer compared with the normal group. (**b**) Low FOXO3 expression was lower in breast cancer based on individual cancer stages. (**c**) Relationship between FOXO3 (204131_s_at probe) expression level and overall survival of patients with breast cancer. (**d**) Relationship between FOXO3 (204131_s_at probe) expression level and relapse‐free survival of patients with breast cancer.

To determine the capacity of MiRNA‐96‐5p on tumorigenesis in breast cancer, transfection of MiRNA‐96‐5p inhibitor into MCF‐7 and T47D was performed. This confirmed that transfection of MiRNA‐96‐5p inhibitor could significantly inhibit MiRNA‐96‐5p in MCF‐7 and T47D cells (Fig 1a,b). Furthermore, proliferation in MCF‐7 cells with transfection MiRNA‐96‐5p inhibitor was obviously suppressed (Fig 1c). The same phenomenon occurred in T47D cells (Fig 1d). After transfection with MiRNA‐96‐5p inhibitor, MCF‐7 and T47D had weaker colony formation ability compared with transfected scramble (Fig 2e,f). These results indicated that MiRNA‐96‐5p had an ability to promote cell proliferation in breast cancer.

### FOXO3 is downregulated in breast cancer and correlated with prognosis

We performed an analysis of FOXO3 expression in breast cancer tissues by using GEPIA and UALCAN databases. Statistical analysis in the UALCAN database revealed low FOXO3 expression in breast cancer compared with the normal group, and the difference was statistically significant (*P* < 0.05), as shown in Fig 2a. Further analysis found that expression of FOXO3 was lower in breast cancer based on individual cancer stages than normal tissues, and the difference was statistically significant (*P* < 0.05), as shown in Fig 2b. In order to further clarify the relationship between FOXO3 expression level and prognosis of breast cancer patients, KM Plotter database analysis showed two survival curves of high FOXO3 expression and low expression groups (different probes), and found that FOXO3 expression level has a significant impact on OS and RFS of patients (Fig 2c,d).

### FOXO3 is a direct target of MiRNA‐96‐5p

**Figure 3:**
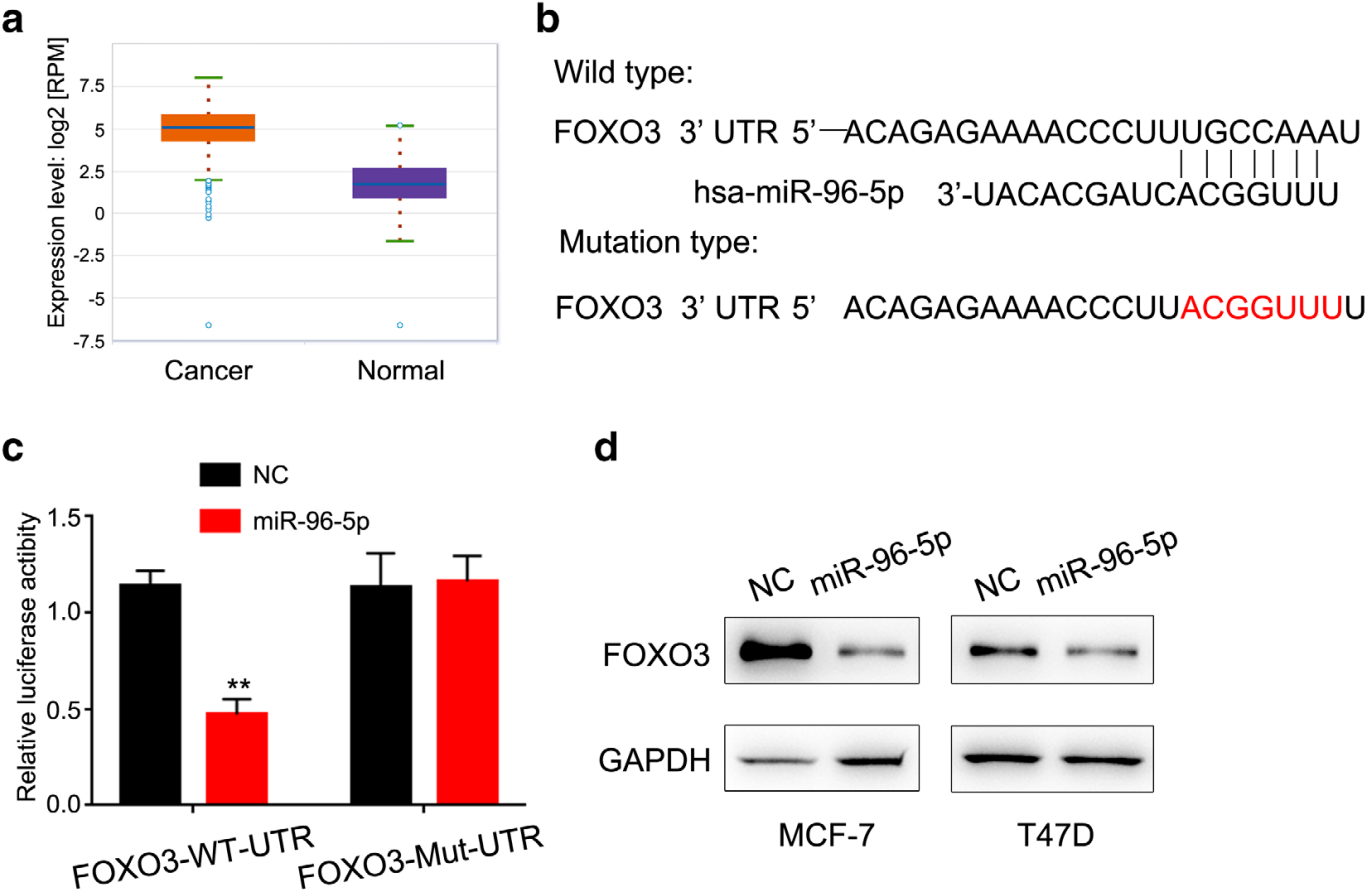
FOXO3 is a direct target of MiRNA‐96‐5p. (**a**) MiRNA‐96‐5p is upregulated in breast cancer tissues as compared to normal tissues analyzed with the starBase v3.0 database. (**b**) Schematic representation of predicted binding sites on miR‐96‐5p that bind to the 3′UTR region of FOXO3 mRNA. The seed sequence of miR‐96‐5p and the mutant mismatched FOXO3 3′UTR are shown. (**c**) 293T cells were cotransfected with Renilla luciferase control (pRL‐TK), wild‐type or mutant FOXO3 3′UTR and pCDNA3‐miR‐96‐5p or the luciferase reporter gene of the control vector. (**d**) Western blot assay showed FOXO3 expression levels in miR‐96‐5p overexpressed breast cancer cells.

To determine which miRNAs can efficiently target FOXO3, we combined the databases (miRBase and Targetscan). We noticed that MiRNA‐96‐5p is one of the candidate genes that contains a 3′UTR sequence matching FOXO3 mRNA (Fig 3b). In addition, we found the expression of MiRNA‐96‐5p was lower in breast cancer tissue (Fig 3a). To determine whether FOXO3 is a direct target of MiRNA‐96‐5p, we cloned the full length 3′UTR sequence of FOXO3 mRNA containing a wild‐type or mutant MiRNA‐96‐5p binding sequence into the firefly luciferase reporter plasmid. We examined the effect of MiRNA‐96‐5p on luciferase activity in 293T cells. The results showed that luciferase activity was significantly inhibited in reporter gene containing wild type 3′UTR of FOXO3 but has no effection in the reporter gene with mismatched binding site (Fig 3c). These data suggest that MiRNA‐96‐5p may inhibit FOXO3 expression through its 3′UTR region binding sequence.

### FOXO3 inhibits miR‐96‐5p‐induced breast cancer cell proliferation

**Figure 4:**
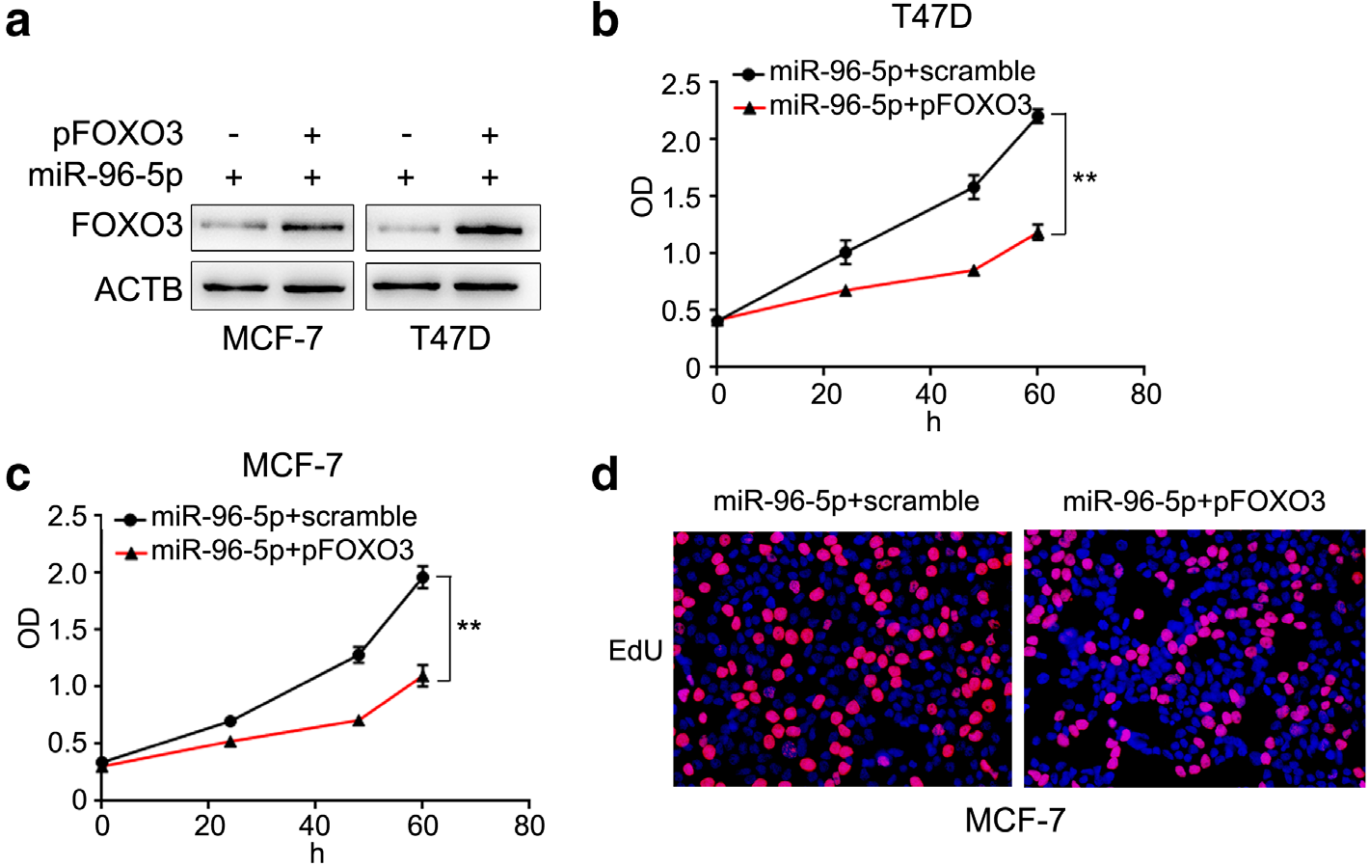
FOXO3 inhibits miR‐96‐5p‐induced proliferation in breast cancer cells. (**a**) Western blot assays revealed that FOXO3 expression obviously upregulated by transfected FOXO3 plasmid into breast cancer cells. (**b**,**c**) Proliferation curves of scramble and pFOXO3 transfected miR‐96‐5p overexpressed breast cancer cells. (**d**) EdU assays of scramble and pFOXO3 transfected miR‐96‐5p overexpressed MCF‐7 cells.

If FOXO3 serves as a functional target of miR‐96‐5p in cancer cell proliferation, re‐expression of FOXO3 in miR‐96‐5p‐overexpressing cells should be able to counter the effects of miR‐96‐5p. To test the hypothesis, we reintroduced FOXO3 into miR‐139‐overexpressing cells (Fig 4a). The result of both CCK8 and EdU assays showed that re‐expression of FOXO3 inhibited the proliferation of miR‐96‐5p‐overexpressing cells (Fig 4b,c,d). These findings indicate that FOXO3 is a functional mediator for miR‐96‐5p on proliferation in breast cancer cells.

## Discussion

The occurrence and development of tumors is a multifactor, multistep, and gradual development process, which include the inactivation of tumor suppressor genes, the activation of oncogenes, abnormal cell cycle regulation, and the formation of tumor blood vessels. In recent years, malignant tumors, including solid tumors and hematological malignancies, have become one of the biggest killers of human health worldwide, especially breast cancer, which is currently the highest incidence of cancer in women worldwide. Infiltration and metastasis of breast cancer are the key factors affecting the efficacy and survival time of patients. The mechanism of metastasis is still unclear, with no effective treatment. Actively exploring the biological mechanism of breast cancer development has practical significance for early diagnosis, reduction of recurrence and metastasis, and new strategies for treating lung cancer.

With large‐scale gene sequencing, it was surprisingly found that less than 2% of the human genome is transcribed into RNA encoding the protein, mRNA. More than 98% of the genes are transcribed into non‐coding protein RNA. Recent studies have found that ncRNA regulates transcriptional processes and post‐transcriptional processes, and plays an important role in the development of important biological processes and diseases. MicroRNAs are non‐coding, small‐molecule single‐stranded RNAs composed of 22 or so ribonucleotides that are highly conserved across species and evolution. MicroRNAs interact with the 3′‐end non‐coding region of mRNA in a complete or incomplete complementary manner, promoting mRNA degradation and inhibiting miRNA translation. MicroRNA has a regulatory effect on many genes, and plays an important role in cell growth and development, differentiation, proliferation, apoptosis, cell cycle and other cell biological behaviors. Some miRNAs have the function of suppressor oncogene, and it can inhibit tumor growth, promote apoptosis.^1^ The Other miRNAs have the function of proto‐oncogenes, and inhibit the the expression of tumor suppressor genes, promote cellproliferation, shorten the cell cycle, and promote cell migration and metastasis.^16^, ^17^, ^18^, ^19^ MiRNA‐96‐5p has been recognized to function as an oncogene. For example, miRNA‐96‐5p interacts with the target gene FOXO1 to inhibit the expression of FOXO1 and promote the proliferation of papillary thyroid cancer cells.^13^ Our study found that miRNA‐96‐5p expression was increased in breast cancer tissues and promoted breast cancer cell proliferation by downregulating the expression of transcription factor FOXO3.

As a member of the forkhead type transcription factor family, FOXO3 is an important tumor suppressor gene in a variety of human cancers, such as liver cancer, and neuroblastoma.^20^ In studies of neuroblastoma, miRNA‐96‐5p plays an important role in tumor cell proliferation, survival, and migration by regulating the expression of FOXO3 and Bcl‐2.^21^ In breast cancer, FOXO3 can be activated by paclitaxel, gefitinib, trastuzumab, leading to increased apoptosis of breast cancer cells, suggesting that activation of FOXO3 can inhibit breast cancer cell proliferation.^22^ In summary, the activation of FOXO3 may become an intervention method for the treatment of malignant tumors.

Our study investigated the biological role of miRNA‐96‐5p in breast cancer. After miRNA‐96‐5p inhibitor transfection, FOXO3 expression was significantly increased, and cell migration and proliferation was reduced. Our results showed that miRNA‐96‐5p is an miRNA that functions as an oncogene, and FOXO3 is a tumor suppressor gene. In breast cancer cells, miRNA‐96‐5p functions as an oncogene by inhibiting FOXO3 expression. Although this experiment verified that miRNA‐96‐5p can regulate the expression of FOXO3, each target genes of miRNA‐96‐5p is still unclear, and further research is needed. Therefore, the effect of miRNA‐96‐5p on the biological behavior of breast cancer cells may be a comprehensive effect. In breast cancer cells, which target genes can be regulated by miRNA‐96‐5p remains unclear, and further research is needed.

## Disclosure

Qin Pan designed the study. Xiang Wang designed the study and wrote the manuscript. Ziyi Yin, Wenyan Wang, Gengbao Qu and Lin Wang performed the study and wrote the manuscript. The authors declare no competing financial interests.
